# The pathogenesis and therapeutic strategies of heat stroke-induced endothelial injury

**DOI:** 10.3389/fcell.2025.1569346

**Published:** 2025-07-09

**Authors:** Shaokang Wang, Xiaoting Zhang, Yongqi Zhang, Nannan Wu, Lulong Bo, Meitang Wang

**Affiliations:** ^1^ Department of Emergency, Changhai Hospital, Naval Medical University, Shanghai, China; ^2^ Faculty of Anesthesiology, Changhai Hospital, Naval Medical University, Shanghai, China

**Keywords:** heat stroke, heat stress, endothelial dysfunction, vascular barrier, glycocalyx

## Abstract

Heat stroke is a severe and life-threatening condition characterized by elevated core body temperature and central nervous system dysfunction, often accompanied by multi-organ damage. The incidence and mortality of heat stroke are increasing due to global warming and more frequent heatwaves. This review aims to summarize the recent progress in understanding the pathogenesis of heat stroke-induced endothelial injury and explore potential therapeutic strategies. The vascular endothelium plays a crucial role in maintaining vascular homeostasis, and its dysfunction is a key factor in the development of heat stroke complications. The pathogenesis of heat stroke-induced endothelial injury involves multiple mechanisms, including degradation of the endothelial glycocalyx, impaired vascular tone regulation, disruption of intercellular junctional proteins, and activation of regulated cell death pathways. Biomarkers such as syndecan-1, endothelin-1, and von Willebrand factor are associated with endothelial injury and can predict disease severity and outcomes. Potential interventions include early fluid resuscitation, heat acclimation, and targeted therapies to inhibit specific cell death pathways or protect the endothelial glycocalyx. Further research is needed to elucidate the detailed mechanisms and develop targeted therapeutic interventions to reduce the morbidity and mortality of heat stroke.

## 1 Introduction

The frequency, duration, and intensity of heat waves have increased worldwide under enhanced global warming, especially over the past two decades (Perkins-Kirkpatrick and Lewis, 2020; Watts et al., 2021). The Lancet-Planet Health shows Globally, 5,083,173 deaths were associated with non-optimal temperatures per year, accounting for 9.43% of all deaths (0.91% were heat-related) (Zhao et al., 2021). Heat stroke (HS) is the most hazardous condition in a spectrum of illnesses progressing from heat exhaustion to heat stroke, clinically characterized by a core temperature of >40.5°C and abnormalities of the central nervous system, including changes in mental status, convulsions or coma and accompanied by life threatening multiple organ damage. The primary pathogenic mechanism of heat stroke involves an imbalance between heat production by and dissipation from the body caused by exposure to a hot environment and/or intense exercise. Depending on its cause and degree of susceptibility within the population, HS may be categorized as either classic (passive) or exertional heat stroke. Although substantial progress has been achieved in the treatment of HS, the mortality of HS is still high. Between 2015 and 2017, the incidence of heat stroke in Japan during the period from June to September was 37.5 cases per 100,000 population (95% CI, 36.8–38.2), and in 2018, it was 74.4 cases per 100,000 population (95% CI, 72.7–76.1) (Ogata et al., 2021). Based on a Japanese cross-sectional survey, out of 763 patients admitted with heat illness (56.9% non-exertional heat stroke and 40.0% exertional heat stroke), the mortality rate was 4.6% (Shimazaki et al., 2020). Comprehending the etiology and pathophysiological mechanisms underlying HS is crucial for identifying prospective therapeutic targets.

The vascular endothelium, comprising endothelial cells (ECs), forms a monolayer barrier along the innermost structure of the arteries, capillaries and veins. Healthy endothelial cells are essential for important physiological functions, including barrier functions, immune response, vascular permeability, coagulation, and angiogenesis (Trimm and Red-Horse, 2023). Damage to ECs in the lungs, brain, heart and liver has been documented in primate models and patients suffered from heat stroke (Abdullah et al., 2024). Heat stress induces ECs injury through direct thermal cytotoxic effects and secondary excessive systemic inflammatory response syndrome. The injury is supported by the finding of increased serum endothelial cell damage biomarkers, such as intercellular adhesion molecular-1 (ICAM-1), soluble endothelial cell leukocyte adhesion molecule 1, endothelin, von Willebrand factor-antigen and endothelial glycocalyx component syndecan-1 (SDC-1) in the heat stroke models of experimental animals and patients (Tong et al., 2014; Umemura et al., 2018). This ECs overactivation impair vascular integrity and barrier function, leading to increased permeability, coagulopathy, and inflammation of heat stroke. The lung emerges as a particularly vulnerable target organ in instances of heat stroke. Impairment of vascular integrity precipitates severe disruptions in the process of gas exchange, which may ultimately culminate in the development of acute lung injury (ALI) and, in more severe cases, progress to acute respiratory distress syndrome (ARDS). Damage and dysfunction to ECs can precipitate injury to other vital organs, such as acute liver injury, acute kidney injury, encephalopathy, and disseminated intravascular coagulation (DIC). During hospital stays, 20% of inpatients require mechanical ventilation and 2% received renal replacement therapy (Kaewput et al., 2021). Consequently, it is of paramount importance to elucidate the specific mechanisms underlying EC dysfunction in the context of heat stroke.

In this review, we summarized the recent research progress on the potential underlying mechanisms and fundamental management principles associated with heat stroke and heat stroke-induced endothelial injury. This comprehensive analysis serves to establish a theoretical foundation and delineate future research directions in the study of heat stroke and its associated endothelial complications.

## 2 Related mechanisms of heat stroke-induced endothelial injury

**FIGURE 1:**
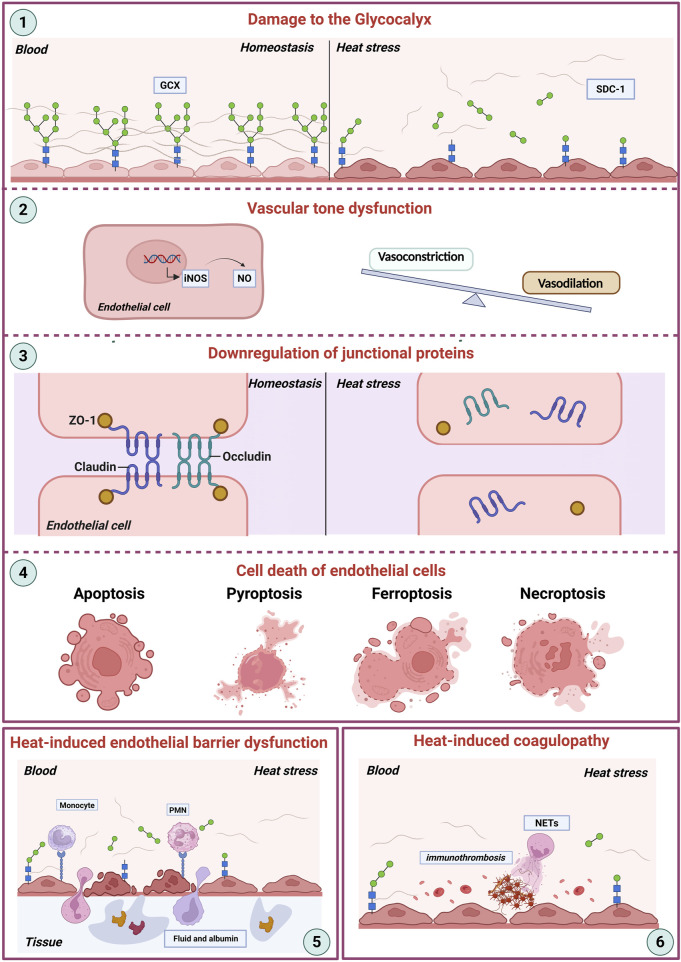
Heat stroke-induced endothelial dysfunction. This schematic illustrates the key mechanisms contributing to endothelial damage during heat stroke. (1) Damage to the Glycocalyx: Heat stress directly disrupts the endothelial glycocalyx (GEX), a critical surface layer maintaining vascular homeostasis between the blood and the vessel wall. (2) Vascular Tone Dysfunction: Damaged endothelial cells show dysregulated nitric oxide (NO) production, often via increased inducible nitric oxide synthase (iNOS) expression. This imbalance disrupts the normal equilibrium between vasoconstriction and vasodilation. (3) Downregulation of Junctional Proteins: Heat stress impairs endothelial barrier integrity by downregulating essential tight junction proteins (ZO-1, Occludin, Claudin), further compromising vascular homeostasis. (4) Endothelial Cell Death: Severe heat stress triggers various pathways of endothelial cell death, including apoptosis, pyroptosis, ferroptosis, and necroptosis. (5) Endothelial Barrier Dysfunction: Cumulative damage encompassing glycocalyx degradation, junctional disruption, and cell death culminates in severe barrier failure, manifesting as increased vascular permeability and extravasation of fluid and albumin into tissues, ultimately contributing to organ dysfunction. (6) Coagulation system: NETs and other inflammatory processes drive pathological clot formation, culminating in disseminated intravascular coagulation (DIC). Abbreviations: GCX, glycocalyx; SDC-1, syndecan-1; iNOS, inducible nitric oxide synthase; NO, nitric oxide; ZO-1, zonula occludens-1. NETs, neutrophil extracellular traps; DIC, disseminated intravascular coagulation.

The endothelial glycocalyx, cell junctions, and intracellular homeostasis are of vital importance for maintaining the structure and function of endothelial cells, as well as preventing the adhesion of inflammatory cells (Vestweber et al., 2009; Foote et al., 2022). The shedding of the glycocalyx, compromised structural-barrier integrity, and regulated cell death of endothelial cell have been observed in the heat stroke model, and all of which contribute the coagulopathy and inflammation associated with heat stroke (Figure 1) (Roberts et al., 2005; Peng et al., 2023).

### 2.1 Damage to the glycocalyx

The glycocalyx is an endovascular barrier structure that covers the surface of endothelial cells. The main components include sulfated proteoglycans (syndecan and glypican), glycosaminoglycans (heparan sulfate, chondroitin sulfate, hyaluronic acid, and dermatan sulfate), and plasma proteins (albumin and antithrombin). It plays important physiological roles, including maintaining barrier function, exerting antithrombotic and anti-inflammatory effects, and regulating vascular permeability. The degradation of the endothelial glycocalyx directly exposes endothelial surface adhesion molecules, including ICAM-1 and vascular cell adhesion molecule-1 (VCAM-1), thereby amplifying leukocyte-endothelial adhesion and subsequent inflammatory infiltration (Petrovich et al., 2016; Van Steen et al., 2023). Concurrently, the liberation of stored chemokines such as interleukin-8 (IL-8) and monocyte chemoattractant protein-1 (MCP-1) activates the NF-κB signaling pathway, resulting in upregulation of pro-inflammatory cytokine expression (Graham et al., 2019; Akhtar and Sharma, 2022). Glycocalyx impairment further disrupts endothelial barrier integrity through abnormal phosphorylation of tight junction proteins, particularly zonula occludens-1 (ZO-1) and occludin (Li J. et al., 2021; Cao et al., 2023).

Experimental evidence indicates that heparan sulfate degradation compromises antithrombin III binding capacity, compromising endogenous anticoagulant mechanisms disrupting endogenous anticoagulant mechanisms and promoting microthrombus formation (Chappell et al., 2014a; Nordling et al., 2015). In addition, pro-inflammatory cytokines stimulate neutrophils to release neutrophil extracellular traps (NETs), these chromatin-DNA-protein complexes entrap platelets and activate coagulation factor XII, thereby amplifying thrombogenesis through reciprocal activation of inflammatory and coagulatory cascades (McDonald et al., 2017; Zhang et al., 2022). These interconnected mechanisms collectively establish a pathological cascade that perpetuates endothelial dysfunction, systemic inflammation, and coagulopathy in heat stroke progression.

The shedding of glycocalyx is mediated by various specific enzymes, with hyaluronidase activity being significantly elevated under heat stress conditions (Clay and Adler, 1967). The degradation of the glycocalyx layer leads to the release of its components, such as SDC-1, heparan sulfate, hyaluronic acid, and chondroitin sulfate, into the plasma. In experimental heat stroke models, the degradation of the glycocalyx layer results in a thinner and more sparse structure, which allows the translocation of plasma proteins and fluid across the vascular wall, thereby contributing to the formation of tissue edema, such as cerebral edema or pulmonary edema. Heat stress induces glycocalyx shedding, as evidenced by fluorescence staining revealing diminished expression of glypican-1 (GPC-1) and SDC-1 in pulmonary vascular endothelia following heat stroke. And ELISA quantification demonstrated elevated plasma levels of both GPC-1 and SDC-1 post-thermal challenge. In heat stroke rat models (ambient temperature: 40°C ± 2°C; relative humidity: 65% ± 5%), pretreatment of heparanase - the enzyme responsible for cleaving heparan sulfate side chains - significantly potentiated heat stress-induced vascular hyperpermeability and exacerbated endothelial dysfunction. This pathological progression was associated with enhanced pulmonary tissue apoptosis, inflammatory responses, and oxidative stress. Notably, therapeutic intervention with unfractionated heparin effectively mitigated thermal stress-mediated glycocalyx damage (Cao et al., 2023). Upon transmission electron microscopy examination of the pulmonary capillaries in heat stroke rats, it was found that the endothelial glycocalyx began to shed following the induction of heat stress, with this shedding becoming more obvious and even absent by 24 h post-induction. Plasma SDC-1 and hyaluronic acid, which are components of the endothelial glycocalyx, gradually increased after the onset of heat stroke and peaked at 24 h. The levels of plasma SDC-1 and hyaluronic acid were positively correlated with the circulating levels of coagulation markers, including von Willebrand factor (vWF), thrombin-antithrombin complex, and plasmin-antiplasmin, and endothelin-1 (ET-1), as well as pro-inflammatory cytokines such as IL-6 and tumor necrosis factor-alpha (TNF-α).

In heat stroke, vascular endothelial cells generated excessive reactive oxygen species (ROS), a key mediator of endothelial glycocalyx degradation. Notably, N-acetyl cysteine protected against endothelial glycocalyx injury by reducing ROS production in heat-stressed vascular endothelial cells (Peng et al., 2023). Inhalation of 2% hydrogen gas significantly mitigated heat stroke-induced shedding of the vascular endothelial glycocalyx in rat models. This protective effect was mediated by its dual mechanisms of antioxidant and anti-inflammatory mechanisms (Truong et al., 2021; Zang et al., 2024). Multiple evidence suggested that bone marrow-derived mononuclear cells (BMDMs) had host-protective paracrine properties, and they were reported to reduce organ dysfunction caused by ischemic reperfusion and systemic inflammation in several animal studies. In a rat model of heat stroke, administration of BMDMs significantly attenuated the elevation of SDC-1 at 6 and 12 h after heat stress. Moreover, BMDMs intervention markedly suppressed serum levels of pro-inflammatory cytokines, including TNF-α and IL-6 (Umemura et al., 2018).

Dexmedetomidine (DEX), a sedative that acts on the α2 adrenergic receptor, is frequently utilized in intensive care settings. Studies have demonstrated that DEX can attenuate the levels of serum inflammatory cytokines in critically ill patients and exhibit anti-inflammatory and anti-apoptotic effect in sepsis. Heat stroke elicits a sepsis-like inflammatory response, and DEX can attenuate heat stroke-induced intestinal barrier disruption in rats, mitigate sepsis-associated inflammation and multi-organ damage, and enhance survival rates by modulating the NF-κB pathway (Xia et al., 2017). Heat stroke leads to a reduction in the glycocalyx thickness, an effect that can be mitigated by the administration of DEX. This intervention also results in a decrease in the elevation of plasma SDC-1 levels induced by heat stroke (Kobayashi et al., 2018).

### 2.2 Vascular tone dysfunction

Vascular tone, defined as the balance between the degree of constriction of the blood vessel and its maximum dilation, is modulated by the release of relaxing and constricting factors derived from the endothelium (Ajoolabady et al., 2024). The endothelium exerts significant influence on the regulation of vascular tone through the synthesis and release of a diverse array of endothelium-derived factors. These factors encompass vasodilatory substances such as prostacyclin and nitric oxide (NO), as well as endothelium-dependent hyperpolarization factors. Additionally, the endothelium also produces endothelium-derived contracting factors, thereby modulating vascular tone in a multifaceted manner (Godo and Shimokawa, 2017; Ajoolabady et al., 2024).)

The mean concentration of NO in heat stroke patients was significantly elevated compared to that in control subjects. Furthermore, non-survivors exhibited higher NO levels than survivors. Additionally, NO concentration demonstrated a positive correlation with the Acute Physiology and Chronic Health Evaluation II (APACHE II) score (Warsy et al., 1999; Cheng and MacDonald, 2019). Under heat stress conditions, elevated NO levels augment cutaneous blood flow, thereby enhancing heat dissipation and providing protection against various heat-related disorders. However, excessive NO production may excessively reduce blood pressure, potentially giving rise to complications (Gostimirovic et al., 2020; Suschek et al., 2022). Nitric oxide synthase (NOS) can be classified into three types: endothelial nitric oxide synthase (eNOS), neuronal nitric oxide synthase (nNOS), and inducible nitric oxide synthase (iNOS), these enzymes facilitate the conversion of l-arginine into citrulline and NO. iNOS has been shown to be upregulated by lipopolysaccharide, IL-1, and TNF-α, all of which are elevated in heat stroke, thereby resulting in the production of substantial quantities of NO (Quirino et al., 2013; Lim, 2018; Snipe et al., 2018).

However, heat stroke is characterized by a paradoxical duality in vascular tone regulation, encompassing both systemic vasodilation for thermolysis and localized vasoconstrictive responses that exacerbate end-organ injury. Notably, clinical and experimental evidence suggests that hyperthermia-induced vasoconstriction of the carotid artery, resulting in cerebral ischemia and subsequent brain damage. *In vitro* studies using rabbit carotid artery strips demonstrated that stepwise heating from 37°C to 47°C elicited reproducible, temperature-proportional contractions in a graded manner. Specifically, heating decreased the contractile responses to norepinephrine and electrical field stimulation but increased contraction in response to KCl. Notably, these effects were not abolished by pretreatment with the neuronal blocker tetrodotoxin (Mustafa et al., 2004; Mustafa et al., 2007). Additionally, ethanol was found to potentiate heat-induced contraction (Mustafa and Ismael, 2017). Clinically, these findings underscore the therapeutic rationale for targeted cooling, particularly of the neck region, to mitigate temperature-sensitive vasoconstrictive responses. However, the interplay between hyperthermia, endothelial injury, and smooth muscle reactivity remains incompletely elucidated, necessitating further research to delineate tissue-specific vascular responses and optimize hemodynamic management in heat stroke.

### 2.3 Downregulation of junctional proteins

The integrity of the endothelial barrier is crucial for maintaining vascular homeostasis and regulating the passage of fluids, solutes, and cells between the bloodstream and tissues. The junctional proteins, including ZO-1, claudin-5, JAM-A, VE-cadherin, and occludin, are essential for maintaining the structural and functional integrity of endothelial cell-cell junctions, and their disruption can have profound pathological consequences.

Heat stress activates various inflammatory pathways, including the p38 MAPK pathway, which plays a pivotal role in endothelial barrier dysfunction. The activation of p38 MAPK leads to the phosphorylation of heat shock protein 27 (HSP27) (Mertenskötter et al., 2013; Huang J. et al., 2022). Which in turn affects the cytoskeletal dynamics and tight junction integrity of endothelial cells. The phosphorylation of HSP27 and other cytoskeletal proteins under heat stress conditions leads to the reorganization of the actin cytoskeleton. This reorganization results in increased endothelial permeability and barrier disruption.

Recent studies have demonstrated that heat stress can induce the expression of protease-activated receptor 1 (PAR1) in endothelial cells and disrupt endothelial barrier function through the activation of the PAR1 signaling pathway. The specific mechanisms involve the release of matrix metalloproteinase-1 (MMP-1) from endothelial cells, leading to the rearrangement of F-actin and actin phosphorylation, which subsequently increases endothelial permeability (Xu et al., 2015a; Zhang et al., 2017). Furthermore, inhibition of PAR1 using specific inhibitors and neutralizing antibodies can significantly reduce heat stress-induced endothelial permeability (Xu et al., 2015b). The increase in endothelial permeability results in pulmonary edema, increased alveolar-capillary permeability, and leukocyte infiltration, which contribute to the development of ALI and ARDS. Additionally, the traditional Chinese medicine Xuebijing injection has been proven to protect endothelial barrier function by inhibiting the PAR1-moesin signaling pathway (Epstein and Yanovich, 2019). These findings highlight the potential therapeutic role of PAR1 inhibitors and traditional Chinese medicine in mitigating heat stress-induced endothelial dysfunction and associated pulmonary complications.

### 2.4 Cell death of endothelial cells

Heat stress, as a physical stimulus, can cause instantaneous and catastrophic cell death when intense. Additionally, the pathogenesis of heat stroke-induced cell death involves multiple mechanisms, including apoptosis, pyroptosis, necroptosis, ferroptosis, and PANoptosis (Shi et al., 2017; Bertheloot et al., 2021; Wang Z. et al., 2024). These regulated cell death pathways play distinct roles in modulating the progression of heat stroke and the associated multi-organ dysfunction at various levels.

Pyroptosis is a form of regulated cell death characterized by the formation of pores in the cell membrane, primarily composed of gasdermin D (GSDMD), leading to cell swelling, lysis, and the release of inflammatory cytokines such as IL-1β and IL-18. Research indicates that the high mobility group box-1 protein (HMGB1) signaling is activated during heat stroke, leading to cell pyroptosis. Elevated levels of HMGB1 are consistently observed in patients with heat stroke who exhibit significantly higher mortality rates (Lu et al., 2004; Tong et al., 2011; Dehbi et al., 2012). Endothelial cells cultured at 43°C exhibit cytoskeletal disorganization and become unclear, accompanied by cell swelling. Heat stress induces the cleavage of caspase-1 from precursor form (procaspase-1) into the active caspase-1. The levels of active caspase-1 increase progressively over a specific temperature range, peaking at 43°C, 4 h after the induction of heat stress. Heat stress-induced pyroptosis in human umbilical vein endothelial cells can be effectively mitigated by siRNA targeting GSDMD (Pei et al., 2018). Exertional heat stroke (EHS) triggers endothelial pyroptosis, leading to HMGB1 release, which enhence macrophage pyroptosis and induces immune dysregulation. Neutralizing HMGB1 antibodies significantly reduce tissue damage and systemic inflammation following EHS (Deng et al., 2018; Yu et al., 2024). Recent studies have demonstrated that exosomes derived from mesenchymal stem cells can mitigate organ damage and reduce inflammation in both cellular and animal models of heat stroke. These therapeutic effects are achieved through the inhibition of the HMGB1/NLRP3 inflammasome activation and the pyroptosis pathway. Additionally, miR-548x-3p plays a crucial role in these processes (Wang et al., 2022; Pei et al., 2023).

Ferroptosis is characterized by increased accumulated iron, lipid peroxidation and ROS levels (Xie et al., 2016; Xie et al., 2021 S.). During heat stroke, iron metabolism is disrupted, characterized by elevated iron concentrations in the damaged hippocampus and increased hepcidin levels. Notably, ferroportin 1 expression is significantly downregulated in the hippocampus (Liu et al., 2019). SIRT1-mediated p53 deacetylation can inhibit ferroptosis and alleviate heat stress-induced lung epithelial cell damage (Chen et al., 2022; Zhu et al., 2024). EHS causes an increase in serum myoglobin concentrations, which can induce acute kidney injury through ferroptosis. Baicalein, a natural flavonoid compound, can mitigate heat stress-induced renal injury by reducing the elevation of ROS caused by heat stress, accompanied by changes in ferroptosis-related proteins (Luan et al., 2023). Recent study suggested that ACSL4 was a key molecule driving rhabdomyolysis following EHS in mice. Evidence includes increased ACSL4, iron accumulation, lipid peroxidation, GPX4 decrease, and reduced survival death with ferroptosis inhibitors. Targeting ACSL4, either genetically or pharmacologically, significantly reduced muscle cell death and rhabdomyolysis biomarkers both *in vitro* and *in vivo*, suggesting ACSL4 inhibition is a promising therapeutic strategy to prevent EHS-induced rhabdomyolysis (He et al., 2022). Currently, research on the impact of heat stress on ferroptosis in endothelial cells remains limited.

**FIGURE 2:**
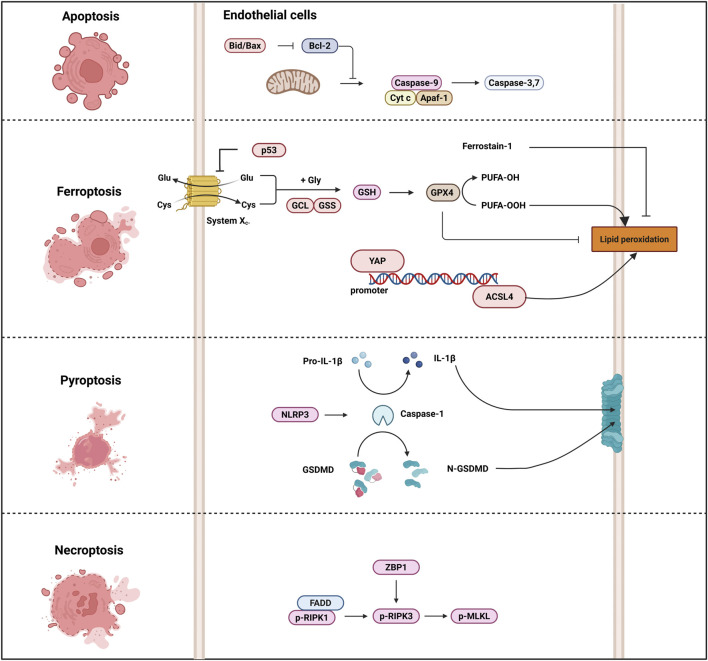
Heat stroke-induced cell death pathway. Several cell death-associated markers are implicated in the pathogenesis of heatstroke. For instance, ACSL4 regulates ferroptosis, caspase-1 and GSDMD execute pyroptosis, and RIPK and MLKL mediate necroptosis. In animal models of heat stroke, therapeutic administration of specific cell death inhibitors attenuates heat-induced organ injury. Excessive activation of these cell death pathways can precipitate cardiovascular collapse, MODS, DIC, and death in heat stroke. Abbreviations: ACSL4, Acyl-CoA synthetase long chain family member 4; GSDMD, Gasdermin-D; RIPK, receptor-interacting protein kinase; MLKL, mixed lineage kinase domain-like protein; MODS, multiple organ dysfunction syndrome; DIC, disseminated intravascular coagulation.

Necroptosis is primarily mediated by the necrosome complex, which includes receptor-interacting protein kinase 1 (RIPK1), RIPK3, and mixed lineage kinase domain-like protein (MLKL) (Gao et al., 2022). In heat stroke, the activation of these proteins leads to the formation of pores in the cell membrane, causing cell swelling and eventual rupture (Yuan et al., 2022; Cai et al., 2023; Li F. et al., 2023; Ai et al., 2024). ZBP1, Z-nucleic acid receptor, was identified to mediate heat stroke via necroptosis by triggering RIPK3-dependent cell death. Deletion of ZBP1, RIPK3, caspase-8, and MLKL can reduce heat stress-induced injuries, including cell death, circulatory failure, and organ damage (Yuan et al., 2022). ROS play a crucial role in heat stroke-induced necroptosis. The clearance of ROS significantly inhibits HS-induced RIPK1/RIPK3-dependent necroptosis both *in vivo* and *in vitro*, thereby alleviating inflammatory responses and multi-organ dysfunction (Li et al., 2020; Xie W. et al., 2021; Huang W. et al., 2022). *In vitro*, pretreatment with necrostatin-1, a RIPK1 inhibitor, attenuated heat stress-induced necroptosis in pulmonary microvascular endothelial cells and inhibited the nuclear-to-cytoplasmic translocation of HMGB1 via the MAPK, NF-κB, and c-Jun signaling pathways (Figure 2) (Huang et al., 2020).

## 3 Roles of endothelial dysfunction in HS

Under heat stress, endothelial cells are subjected to various challenges, including oxidative stress, barrier dysfunction, and cell death, which collectively impair their normal function. The endothelial dysfunction not only affects local blood flow but can also contribute to systemic complications such as multi-organ failure and cardiovascular diseases. Patient mortality with multiple organ dysfunction syndrome reaches 85%, with the respiratory system as the most commonly affected organ system at 85.7% (Varghese et al., 2005). Full understanding of the pathogenesis of endothelial injury requires knowledge of the host thermoregulatory and heat stress responses to extreme heat stress. In addition, understanding the roles of endothelial dysfunction in heat stress is crucial for developing effective interventions to mitigate the adverse effects of heat exposure on human health.

### 3.1 Role of heat-induced endothelial barrier dysfunction

Endothelial barrier dysfunction is intricately linked to the development and progression of various diseases, including ALI, ARDS, and atherosclerosis. Recent advancements in the understanding of heat stress mechanisms have unveiled that heat stress can disrupt endothelial barrier function through multiple signaling pathways and molecular mechanisms. This disruption is a crucial factor in the pathogenesis of heat stroke.

The development of heat stroke involves a transition from a compensatory phase, where thermoregulatory mechanisms attempt to maintain core body temperature, to a decompensatory phase where heat production exceeds heat dissipation. During the compensatory phase, the body attempts to dissipate heat through mechanisms such as vasodilation of cutaneous blood vessels and increased sweating. However, as heat stress continues, the circulatory system becomes overwhelmed, leading to a reduction in circulating blood volume and a subsequent decrease in cardiac output. When the heart can no longer meet the demands of thermoregulation, heat stroke ensues (Epstein and Yanovich, 2019). Heat stress disrupts the integrity of endothelial tight junctions, leading to increased permeability and leakage of plasma proteins. This results in a reduction of intravascular volume and the accumulation of fluid in tissues, causing edema. In addition, heat stress induces endothelial injury, leading to increased vascular permeability, reduced blood volume, tissue edema, and the migration of inflammatory cells. These changes further exacerbate circulatory failure and multi-organ dysfunction.

### 3.2 Role of heat-induced coagulopathy

In severe heat stroke cases, clinical studies have reported a 48% incidence of DIC as a life-threatening complication (Huisse et al., 2008; Hemmelgarn and Gannon, 2013; Hifumi et al., 2018). Critically, patients who develop DIC exhibit significantly higher rates of multiorgan dysfunction and heat stroke-related mortality compared to those without coagulopathy. The pathophysiology of heat-induced coagulopathy is complex and not fully understood. However, it is believed that heat stress can trigger the release of various procoagulant factors, such as vWF and factor VIII, from vascular endothelial cells. These factors promote intravascular thrombosis by enhancing platelet aggregation and activation (da Cruz et al., 2019). Additionally, heat stress can lead to endothelial dysfunction, characterized by the loss of anticoagulant properties and increased expression of adhesion molecules, further contributing to the prothrombotic state. Inflammatory activation also plays a crucial role in the development of heat-induced coagulopathy. Heat stress can induce the release of pro-inflammatory cytokines, such as IL-6 and TNF-α, which in turn activate the coagulation system (Pennings et al., 2022). This interaction between inflammation and coagulation creates a self-amplifying loop, leading to the formation of microthrombi and further endothelial damage (Sylman et al., 2015). Despite its clinical significance, the precise pathophysiological mechanisms underlying heat stroke-induced coagulopathy remain incompletely characterized. Further mechanistic investigations are necessary to identify potential therapeutic targets.

## 4 Biomarkers and potential interventions for endothelial injury in HS

### 4.1 Biomarkers for endothelial injury in HS

As mentioned above, the glycocalyx plays multiple roles in vascular homeostasis. Glycocalyx fragments shed into the bloodstream during sepsis may serve as clinically relevant biomarkers (Uchimido et al., 2019). Numerous preclinical and clinical investigations have established a significant correlation between pro-inflammatory cytokines and biomarkers indicative of glycocalyx degradation. In addition, preclinical and clinical investigations have demonstrated that hypervolemia triggers the secretion of atrial natriuretic peptide from cardiac atrial chambers, a response mediated by mechanical wall stress secondary to atrial distension. Emerging evidence implicates hypervolemia as a contributory factor in glycocalyx degradation through mechanotransduction pathways (Chappell et al., 2014b; Sukudom et al., 2024). Emerging studies reveal pathognomonic elevation of glycocalyx degradation products (specifically SDC-1, heparan sulfate, and hyaluronan) in both experimental heat stroke models and human patient plasma. These circulating biomarkers demonstrate significant associations with multiple clinical parameters, including Sequential Organ Failure Assessment scores, inflammatory cytokine levels, and mortality rates (Kobayashi et al., 2018; Umemura et al., 2018; Truong et al., 2021; Cao et al., 2023; Peng et al., 2023).

Other indicators of endothelial cell activation and compromised integrity include the detachment of endothelial cells and their subsequent release into the peripheral circulation. An increasement in circulating endothelial cells has been observed in an experimental heat stroke rat model, indicating its potential utility as a marker for endothelial injury (Tong et al., 2014).

Endothelins, predominantly ET-1, are potent vasoactive peptides synthesized by endothelial cells that regulate vascular tone and tissue perfusion through endothelin A/endothelin B receptor-mediated signaling. While essential for hemodynamic homeostasis, pathological ET-1 overproduction under stress conditions (e.g., hyperthermia, inflammation) drives microvascular dysfunction, endothelial hyperpermeability, and multiorgan injury, positioning it as both a pathophysiological mediator and therapeutic target in critical illnesses (Banecki and Dora, 2023; Kanai and Clouthier, 2023; Schiffrin and Pollock, 2024). Experimental studies in rodent heat stroke models demonstrate significant elevation of circulating ET-1 levels, with mechanistic analyses revealing that selective ET-1 receptor antagonism ameliorates thermoregulatory failure by attenuating TNF-α production. This therapeutic intervention preserves hemodynamic stability by maintaining mean arterial pressure within physiological thresholds and normalizing cerebral perfusion parameters during heat stroke, suggesting its potential for mitigating blood-brain barrier disruption in hyperthermia-induced encephalopathy (Liu et al., 2004; Chen et al., 2023; Xu et al., 2023).

The pathogenesis of heat stroke is characterized by a significant elevation in biomarkers of endothelial injury, particularly vWF, plasminogen activator inhibitor-1 and soluble thrombomodulin (TM), which are prominently increased in plasma during heat stroke (Bergonzelli and Kruithof, 1991; Shieh et al., 1995; Rücker et al., 2006; Kawasaki et al., 2014). These biomarkers exhibit a strong correlation with the severity of coagulopathy and mortality risk, reflecting the dual activation of thrombo-inflammatory pathways and failure of anticoagulant mechanisms (Shieh et al., 1995; Roberts et al., 2008; Li Y. et al., 2023; Wan et al., 2023). TM confers both anticoagulant and anti-inflammatory properties, thereby enhancing the survival outcomes in patients with septic shock. The administration of thrombomodulin has been shown to alleviate heat stroke-induced multi-barrier dysfunction by mitigating intestinal hyperpermeability and blood-brain barrier disruption. This therapeutic effect is achieved through the suppression of complement activation, endotoxin release, and HMGB1 protein (Hagiwara et al., 2010; Kawasaki et al., 2014; Lin et al., 2024). In patients or animal models of heat stroke, elevated levels of HMGB1, extracellular histone H3, and circulating heat shock proteins (e.g., HSP70) have been observed in plasma (Geng et al., 2015; Schlader et al., 2022). These non-endothelial-specific markers of injury originate from necrotic cells, neutrophil extracellular traps, and stressed parenchymal cells. Collectively, they amplify systemic inflammation through the activation of TLR4/RAGE and NLRP3 inflammasome pathways (Dehbi et al., 2010; Dehbi et al., 2012; Bruchim et al., 2016; Bruchim et al., 2017; Li Y. et al., 2021; Du et al., 2022; Yin et al., 2022).

### 4.2 Potential interventions for endothelial injury in HS

Heat acclimatization is a physiological adaptation process that occurs in response to repeated heat exposure. This process involves a series of physiological changes in the body, including improved cardiovascular function, enhanced sweating efficiency, and better thermoregulation. It was reported that heat acclimatization can enhance the viability and resilience of vascular endothelial cells following heat stress. It also partially restores the normal function of these cells. This protective effect is likely associated with the upregulation of HSP70 expression (Wen et al., 2024).

During passive heat stress, significant augmentation of cutaneous blood flow is primarily supported by a coordinated increase in cardiac output and a concomitant decrease in vascular conductance within non-cutaneous vascular beds (Crandall and Wilson, 2014; Cramer et al., 2022). However, this reflex cutaneous vasodilation is markedly attenuated in healthy older adults (≥60 years), a phenomenon attributed to impairments in both central sympathetic outflows directed to the cutaneous circulation and cutaneous microvascular endothelial function (Kriebel-Gasparro, 2024; Kriebel-Gasparro, 2024). Notably, these age-related deficits may be exacerbated by hypercholesterolemia - particularly elevated low-density lipoprotein levels, a well-established risk factor for atherosclerotic cardiovascular disease. Experimental evidence demonstrates that hypercholesterolemia independently associates with dysregulated sympathetic control and impaired microvascular endothelial responsiveness (Eikelis et al., 2017). In experimental settings using whole-body heating via water-perfused suits to simulate thermal stress, researchers have identified that the diminished reflex vasodilation in older adults result from combined dysfunction in both central sympathetic regulation and peripheral microvascular mechanisms. Importantly, while older adults with untreated hypercholesterolemia demonstrate significant impairment in heat-induced cutaneous vasodilation, this deficit is normalized in age-matched counterparts receiving statin therapy. This therapeutic effect suggests that statins may mitigate cholesterol-related microvascular dysfunction through lipid-independent pleiotropic mechanisms (Greaney et al., 2019).

Heat stroke-induced thermoregulatory deficits and mortality may arise from ischemic and oxidative damage to the hypothalamus, the primary thermoregulatory center in the mammalian brain (Cramer et al., 2022). Compared to normothermic controls, mice exposed to heat stress exhibit significantly elevated levels of cellular ischemia markers (such as glutamate and the lactate-to-pyruvate ratio) in the hypothalamus (Chang et al., 2007; Chen et al., 2009; Zhang et al., 2023). Studies demonstrated that heat stroke triggers intestinal villi injury accompanied by enhanced epithelial cell apoptosis and elevated HIF-1α expression. The intervention of HIF-1α overexpression can suppress eIF2α/ATF4/CHOP pathway, ultimately attenuating heat stroke-mediated intestinal damage. Hypobaric hypoxia preconditioning is a temporary condition triggered by short periods of sublethal hypoxia, which activate various endogenous atrophic signals and provide strong protection against future lethal challenges (Liu et al., 2021; Torosyan et al., 2021). Twenty minutes after heat stress, rats exposed to heat without hypoxia preconditioning (HHP) treatment showed significantly higher core and hypothalamic temperatures, hypothalamic MMP-9 levels, and counts of apoptotic neurons in the hypothalamus, compared to control rats not exposed to heat. These heat-exposed rats also exhibited significantly lower mean blood pressure, hypothalamic blood flow, and PaO_2_ values. However, in rats exposed to heat, HHP significantly increased the hypothalamic levels of HIF-1α, HSP-72, and HO-1, and effectively alleviated hypothalamic hyperthermia, hypotension, hypothalamic ischemia, hypoxia, neuronal apoptosis, and degeneration (Chen et al., 2009; Chao et al., 2020). Once hydroxylated by prolyl hydroxylase, HIF-1α undergoes rapid degradation via the ubiquitin-proteasome pathway (Kim and Yang, 2015). Roxadustat, a hypoxia-inducible factor prolyl hydroxylase inhibitor, can enhance the production of endogenous erythropoietin to treat anemia in chronic kidney disease patients through the activation of the HIF-1α pathway (Chen et al., 2019). Roxadustat pretreatment significantly enhanced renal function, thermotolerance, and survival rate in mice subjected to heat stroke. This protective effect is attributed to the activation of BNIP3-mediated mitophagy, which shields renal tubular epithelial cells from inflammation and apoptosis. Conversely, genetic ablation of BNIP3 not only weakened roxadustat-induced mitophagy but also nullified the renal protective effects mediated by roxadustat (Wang L. et al., 2024).

## 5 Conclusion

Heat stroke-induced endothelial injury is a complex pathological process involving multiple mechanisms, including glycocalyx degradation, vascular tone dysfunction, junctional protein disruption, and regulated cell death pathways. These changes contribute to systemic complications such as multi-organ failure and cardiovascular diseases. Understanding the detailed mechanisms underlying endothelial dysfunction in heat stroke is crucial for developing targeted therapeutic strategies. Potential interventions, such as early fluid resuscitation, heat acclimation, and pharmacological agents targeting specific pathways, show promise in mitigating heat stroke-induced endothelial injury and improving patient outcomes. Future research should focus on further elucidating the pathophysiological mechanisms and identifying more effective biomarkers and therapeutic targets to reduce the high mortality and morbidity associated with heat stroke.

## Abbreviations

HS, heat stroke; EC, endothelial cell; ICAM-1, intercellular adhesion molecular-1; SDC-1, syndecan-1; ALI, acute lung injury; ARDS, acute respiratory distress syndrome; DIC, disseminated intravascular coagulation; VCAM-1, vascular cell adhesion molecule-1; IL, interleukin; ZO-1, zonula occludens-1; vWF, von Willebrand factor; BMDMs, Bone marrow-derived mononuclear cells; DEX, dexmedetomidine; ET-1, endothelin-1; TNF-α, tumor necrosis factor-alpha; ROS, reactive oxygen species; NO, nitric oxide; NOS, nitric oxide synthase; HMGB1, high mobility group box-1 protein; HSP, heat shock protein; GCX, glycocalyx.
